# Transcriptome analysis of eutopic endometrium in adenomyosis after GnRH agonist treatment

**DOI:** 10.1186/s12958-021-00881-3

**Published:** 2022-01-12

**Authors:** Jiao Tian, Nannan Kang, Junxia Wang, Haixiang Sun, Guijun Yan, Chenyang Huang, Jie Mei

**Affiliations:** 1 Reproductive Medicine Center, Nanjing Drum Tower Hospital, The Affiliated Hospital of Nanjing University Medical School, Nanjing, 210008 People’s Republic of China; 2 Center for Molecular Reproductive Medicine, Nanjing University, Nanjing, 210008 China

**Keywords:** Adenomyosis, Gonadotropin-releasing hormone agonist, Chemokine ligand 21, Endometrial receptivity

## Abstract

**Background:**

Adenomyosis is a chronic gynecological disease characterized by invasion of the uterine endometrium into the muscle layer. In assisted reproductive technology (ART), gonadotropin-releasing hormone agonist (GnRHa) is often used to improve pregnancy rates in patients with adenomyosis, but the underlying mechanisms are poorly understood.

**Methods:**

Eutopic endometrial specimens were collected from patients with adenomyosis before and after GnRHa treatment in the midsecretory phase. RNA sequencing (RNA-Seq) of these specimens was performed for transcriptome analysis. The differentially expressed genes (DEGs) of interest were confirmed by real-time PCR and immunohistochemistry.

**Results:**

A total of 132 DEGs were identified in the endometrium of patients with adenomyosis after GnRHa treatment compared with the control group. Bioinformatics analysis predicted that immune system-associated signal transduction changed significantly after GnRHa treatment. Chemokine (C-C motif) ligand 21 (CCL21) was found to be highly expressed in the eutopic endometrium after GnRHa treatment, which may be involved in the improvement of endometrial receptivity in adenomyosis.

**Conclusion:**

This study suggests that molecular regulation related to immune system-associated signal transduction is an important mechanism of GnRHa treatment in adenomyosis. Immunoreactive CCL21 is thought to regulate inflammatory events and participate in endometrial receptivity in adenomyosis.

## Background

Adenomyosis is defined by the presence of endometrial glands and stroma within the myometrium and diffuse enlargement of the uterus. It preferentially affects parous women between the ages of 35 and 50 years, and its incidence range is estimated at 8–27% [1]. Infertile reproductive failures occur in approximately 22% of adenomyosis patients [2]. In vitro fertilization-embryo transfer (IVF-ET) has become the primary method used to treat adenomyosis-associated infertility [3]. In general, practice, gonadotrophin-releasing hormone agonist (GnRHa) is commonly applied in the treatment of adenomyosis patients with assisted reproductive technology (ART). It has been suggested that GnRHa treatment before frozen embryo transfer (FET) is associated with an increased rate of clinical pregnancy in adenomyosis patients [4, 5]. GnRHa treatment is also associated with a dramatic improvement in clinical pregnancy rates in adenomyosis patients with recurrent implantation failure (RIF) [6]. GnRHa has been found to markedly reduce the inflammatory reaction and angiogenesis of ectopic foci in women with adenomyosis, which might have an effect at the tissue level and be involved in the regression of adenomyosis [7]. However, information regarding the effect of GnRHa on endometrial receptivity in women with adenomyosis is unclear.

Thus, elucidating the systemic role and underlying cellular regulatory mechanisms of GnRHa treatment in adenomyosis is necessary to identify impaired endometrial receptivity in adenomyosis-associated infertility.

Therefore, in the present study, RNA sequencing (RNA-Seq) of the eutopic endometrium from GnRHa-treated or untreated patients with adenomyosis was conducted. Differentially expressed genes (DEGs) and molecular pathways/networks enriched in the eutopic endometrium after GnRHa treatment were identified at the transcriptome level. Furthermore, the adenomyosis mice model were employed to explore the function of GnRHa and identified gene CCL21 on the endometrial receptivity. The expression level of the endometrial receptivity marker HOXA10 was significantly increased after combined administration of GnRHa and CCL21 in the uterus of mice with adenomyosis.

## Methods

### Subjects and sample collection

The subjects recruited into the study were women of reproductive age attending the Reproductive Medicine Center of Nanjing Drum Tower Hospital with the complaint of infertility due to adenomyosis from February 2015 to June 2019. Written informed consent was obtained from patients before sampling, and endometrial biopsies were taken solely for research purposes. The study protocol was approved by the Research Ethics Committee of the Nanjing Drum Tower Hospital. None of the patients received oral contraceptives or any other therapy within 3 months prior to sampling, and none experienced complications related to pelvic inflammatory disease, autoimmune diseases or RIF. Adenomyosis in all of the women was diagnosed by ultrasonography and magnetic resonance imaging (MRI). Eutopic endometrium samples were obtained from all 9 women with adenomyosis, and endometrial samples were taken again after treatment with GnRHa (Decapeptyl, triptorelin acetate, 3.75 mg, Ferring GmbH, Germany) for a variable period of 3–6 months before frozen embryo transfer (FET). All the patients had moderate focal adenomyosis of the outer myometrium (severity scores ranging from 4 to 6) according to the transvaginal ultrasound-based “sonographic classification of adenomyosis” [8]. The phase of the menstrual cycle was determined by follicular monitoring under ultrasound. All the samples used for in vitro experiments were collected during the midsecretory phase (LH + 7 of the menstrual cycle) using an endometrial curette. Samples of at least 300 mg were collected under sterile conditions and divided into three parts. The first part was immediately fixed in 4% v/v paraformaldehyde for immunofluorescence studies, and the second part was preserved in a cryotube and stored at − 80 °C. The remaining part was transported to the laboratory on ice in Dulbecco’s modified Eagle’s medium (DMEM)/F-12 medium (Gibco, Grand Island, NY, USA).

### RNA-Seq and data analysis

Total RNA was extracted using TRIzol reagent (Life Technologies, Carlsbad, CA, USA) according to the manufacturer’s protocol. RNA purity and quantity were evaluated using a NanoDrop 2000 spectrophotometer (Thermo Fisher Scientific, Waltham, MA, USA). RNA integrity was assessed using the Agilent 2100 bioanalyzer (Agilent Technologies, Santa Clara, CA, USA). Then, libraries were constructed using the TruSeq Stranded mRNA LT Sample Prep Kit (Illumina, San Diego, CA, USA) according to the manufacturer’s instructions. Transcriptome sequencing and analysis were conducted by OE Biotech Co., Ltd. (Shanghai, China).

Raw mRNA-seq data were trimmed using Trimmomatic v.0.33. The clean reads were mapped to the human genome (GRCh38) using HISAT2 [9]. Fragments per kilobase per million (FPKM) of each gene were calculated using Cufflinks. The coverage rates of transcripts were obtained by calculating the FPKM based on the length of the transcript and the number of reads mapped to the gene/transcript. mRNAs with an absolute Log_2_Ratio ≥ 1 and *P*-value < 0.05 were marked as significant. Hierarchical cluster analysis of DEGs was performed to explore gene expression patterns. GO enrichment and Kyoto Encyclopedia of Genes and Genomes (KEGG) pathway enrichment analyses of the DEGs were performed using R based on the hypergeometric distribution.

### Quantitative reverse transcription PCR (RT-qPCR)

RT-qPCR was performed to validate the gene expression data obtained from deep sequencing. Total mRNA was extracted using TRIzol reagent (Life Technologies, Carlsbad, CA, USA) according to the manufacturer’s instructions. First-strand cDNA was synthesized using primers designed in our laboratory (Table 1). The RT product was amplified using SYBR Green on a 7500 Real-Time PCR System (Thermo Fisher Scientific Inc. Waltham, MA, USA). All samples were run in triplicate, and relative gene expression was analyzed according to the 2^-ΔΔCt^ method. The housekeeping gene Actb was used for normalization of the expression data.

**Table 1:** RT–qPCR primers used in the study

Gene symbol	Forward primer (5′–3′)	Reverse primer (5′–3′)
RAB7B	CTCATTATCGTCGGAGCCATTG	AGTGTGGTCTGGTATTCCTCATA
GALNT13	TTGCCCTTAATAGAAGTCTGCCA	TGGGGAACGATTTATCACACTG
CFD	GACACCATCGACCACGACC	GCCACGTCGCAGAGAGTTC
LHFPL3	CCTACTGGATAGGCGACGG	GAGGCCGATAAAGAAGGAGGC
PLAC9	GAGCACAGCGTGTGACAGA	GATCCACGGTCTTCTCTACCA
SCARA5	AAAGCTATGTACCTACACACCGT	CCGCCGTTTGTGACATGGA
EBF1	AAAGCATCCAACGGAGTGGAA	GCCCTGTCTGTCGTAGAGG
CCL21	GTTGCCTCAAGTACAGCCAAA	AGAACAGGATAGCTGGGATGG
EPHA7	AGAACTATACCCCGATACGAACA	TGGAAATCCAGTTAGTCCGCA
PCDH10	TGGATGGTGGAAGGAGTCTTT	TTCAGCGATATTCCCCACGAA
THEM4	GAACAAGGACCTAAGACTGCTC	AGAACATCACGTATTCAAAGCCC
PPP2R3B	TCCGCAGGGACGAGAGTAG	AAGGTCGGAATGCTTTGGCTC
ACTB	CATGTACGTTGCTATCCAGGC	CTCCTTAATGTCACGCACGAT

Note: ACTB (β-actin) was used as an internal control

Abbreviation: *RT-qPCR* quantitative reverse transcription PCR

### Immunohistochemistry

Patient endometrial tissue was fixed with 10% formalin at room temperature overnight and embedded in paraffin after dehydration with an ethanol gradient (70, 80, 90, 95, and 100%). The endometrial tissue was sliced into 3-μM-thick sections, deparaffinized with xylene, and then hydrated in an ethanol gradient. After the sections had been permeabilized with 1% Triton X-100 for 10 min, antigen retrieval was performed using citrate buffer (pH 6.0) at 120 °C under high pressure for 15 min. The sections were blocked with goat serum for 30 min at room temperature and then incubated with a primary antibody against chemokine (C-C motif) ligand 21 (CCL21) (1:100, ab231116, Abcam) at 4 °C overnight. The sections were rinsed with TBST and incubated with goat anti-rabbit secondary antibody for 1 h at room temperature. The sections were counterstained with hematoxylin after staining with DAB. A nonspecific rabbit IgG was used as a negative control, and the other steps were the same as those described for the experimental group; nonspecific staining was detected in the negative control.

### Western blotting

Mouse uterine tissue was ground and lysed with RIPA buffer (50 mM Tris-HCl, pH 7.6; 150 mM NaCl; and 1.0% NP-40) containing protease inhibitors (Roche, Basel, Switzerland) at 4 °C for 30 min. The protein concentration was measured with a BCA protein assay reagent (Thermo Fisher Scientific, Waltham, MA, USA). The proteins were electrophoresed through a 10% SDS-PAGE gel and transferred to a PVDF membrane (Millipore, Billerica, MA, USA). Antibodies against Integrin β3 (1:2000, BS3660, Bioworld), FOXO1 (1:1000, 2880S, CST), HOXA10 (1:500, sc-271,953, Santa Cruz) and GAPDH (1:10000, AP0063, Bioworld) were used to detect these proteins.

### Animals

All animal experiments were conducted under the guidance of the Laboratory Animal Management Committee (Jiangsu Province, China) and approved by the Institutional Animal Care and Use Committee of Nanjing Drum Tower Hospital (SYXK 2014–0052). ICR mice were raised in the Experimental Animal Center of Nanjing Drum Tower Hospital (Nanjing, China) in a controlled environment at 20 ± 2 °C under SPF-free conditions on a 12:12-h light:dark cycle with a humidity of 50–70% were freely provided food and water. The newborn mice were fed a peanut oil/lecithin/condensed milk mixture (volume ratio 2:0.2:3) in 5 μl (g•body weight) containing 2.7 μmol (kg•body weight) tamoxifen (Shanghai Fudan Forward Pharmaceutical Co, Shanghai, China) every day from days 2–5 after birth to create the adenomyosis model, while mice in the control group were fed vehicle only. Mice in the adenomyosis group were intraperitoneally injected with 8 mg of GnRHa at the age of 3 months. After 4 weeks, the mice treated with GnRHa were mated and infused with CCL21 (58025-M08B, Sino Biological, Beijing, China) recombinant protein into the uterine cavity dpc 1 (the day the vaginal plug formed was day postcoitus 1). The mice were sacrificed on dpc 4 to verify the adenomyosis model and detect endometrial receptivity and Treg cell expression levels.

## Results

### RNA-Seq analysis and identification of DEGs

**Fig. 1:**
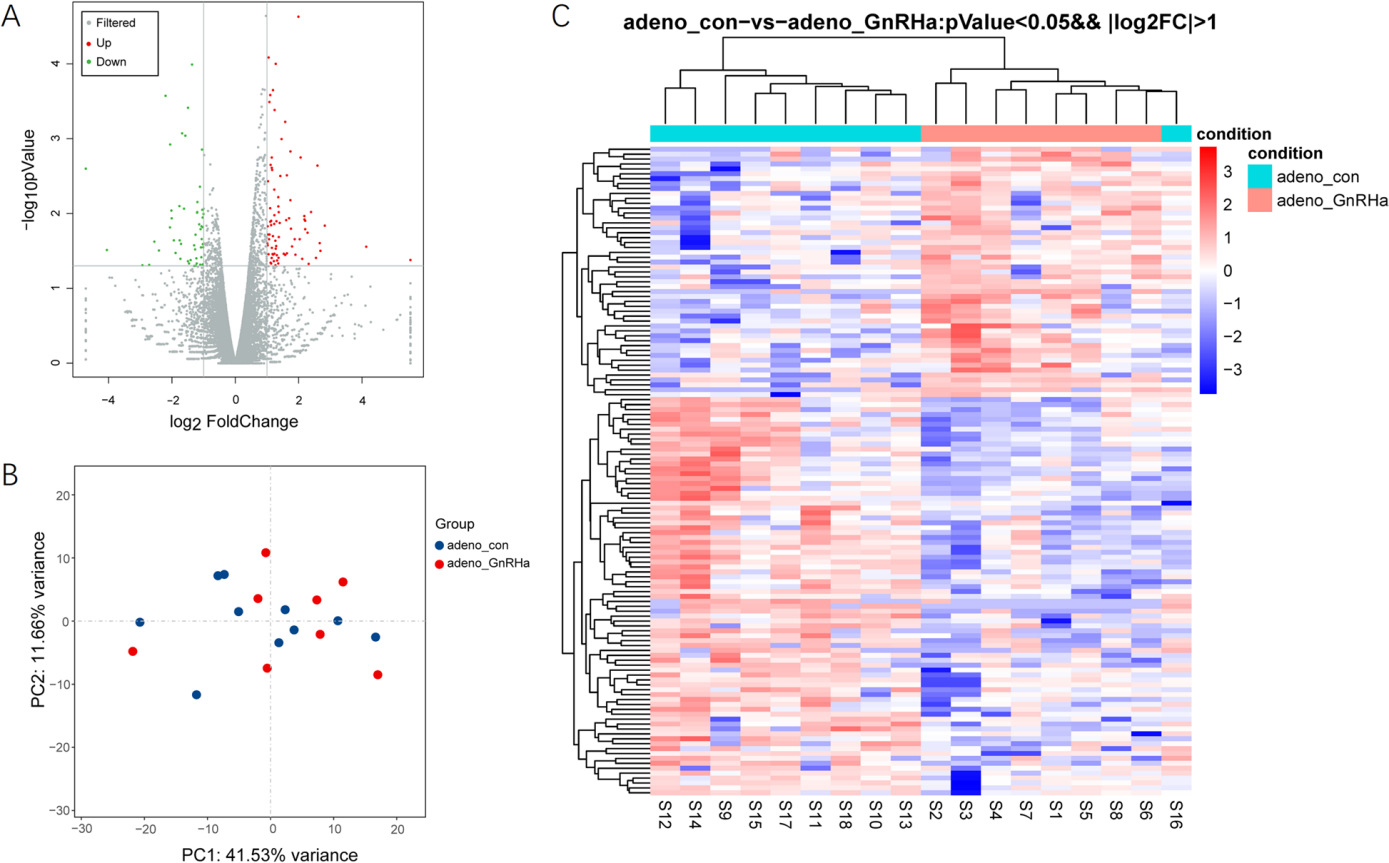
DEGs between the eutopic endometrium from patients with adenomyosis treated with GnRHa and matched adenomyosis endometrium samples were identified by RNA-Seq. **A**, Volcano plots of genes with differential expression. The x axis represents the log_2_ (fold change), and the y axis represents −log_10_ (*P* value), which was calculated by Student’s t test. The red points represent the identified genes with a statistically significant difference in expression (*P* < 0.05 and fold change ≥2). **B**, Principal component analysis (PCA). **C**, Heatmap of genes with differential expression

To better understand the effect of GnRHa in adenomyotic endometrial tissue, we conducted a comparative transcriptomic analysis of the endometrium in 9 patients before and after GnRHa treatment. We obtained an average of 46 million read pairs from each sample and further compared the expression profiles in the endometrium between the GnRHa-treated and control groups. In total, 20,030 annotated mRNAs were identified, and 132 mRNAs (*P-*value < 0.05; absolute Log_2_Ratio ≥ 1) were significantly deregulated (Fig. 1A). Among the identified DEGs mentioned above, 81 genes were upregulated, and 51 genes were downregulated in eutopic endometrium from patients with adenomyosis compared to that from the control subjects.

Principal component analysis (PCA) and hierarchical clustering were performed with the differentially expressed mRNAs. The PCA results illustrated that the GnRHa-treated endometrium from patients with adenomyosis exhibited distinct gene expression profiles compared with those of the control endometrium (Fig. 1B). A heatmap of the DEGs between the GnRHa-treated and control endometrium indicated the profound impact of GnRHa treatment on eutopic endometrial mRNA expression in adenomyosis patients (Fig. 1C).

### Functional analysis of DEGs

**Fig. 2:**
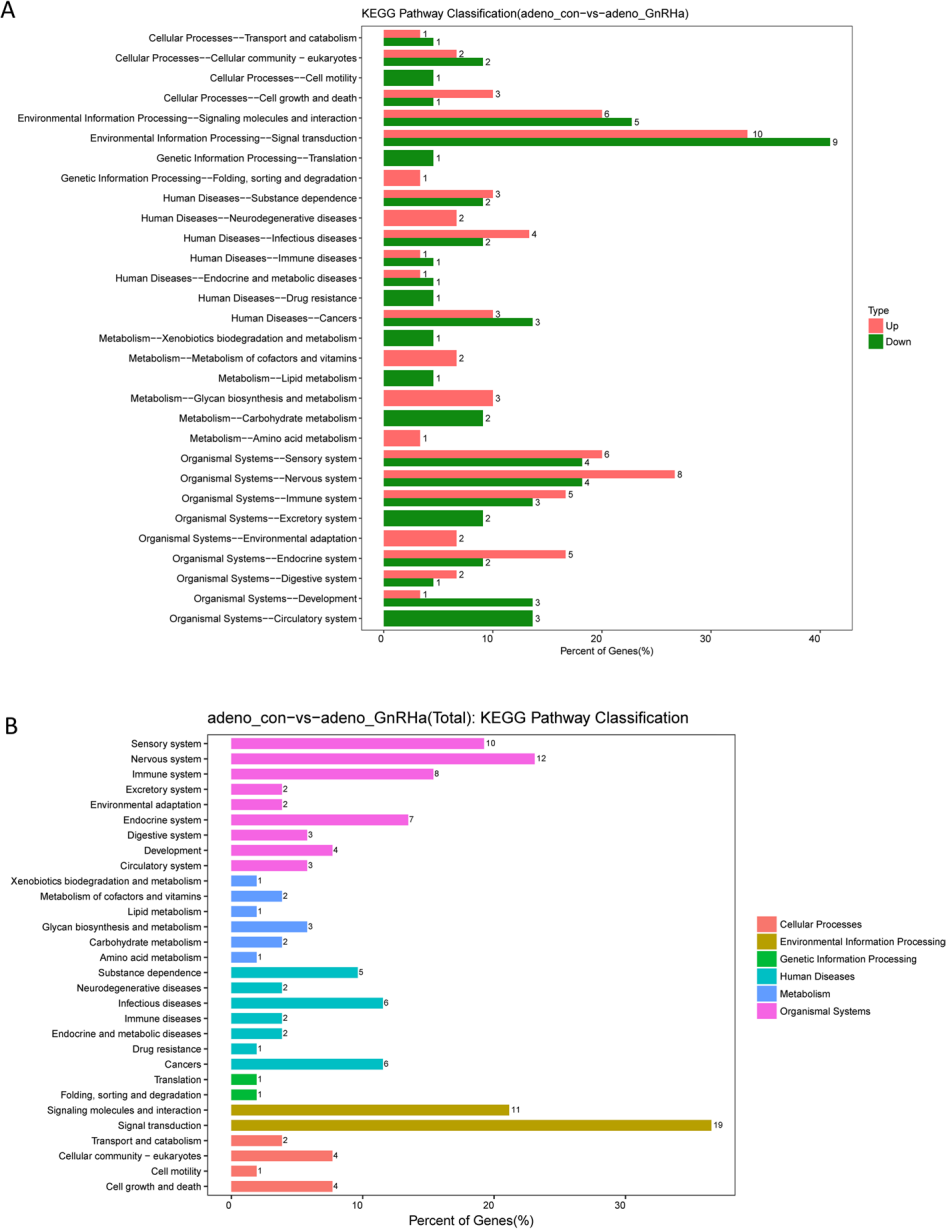
Functional analysis of the DEGs in the endometrium from patients with adenomyosis before and after GnRHa treatment

We performed a functional investigation through KEGG pathway analysis, which identified biological processes significantly enriched in the DEGs (Fig. 2). From the perspective of diseases and disorders, the nervous system and signal transduction were the top two significantly enriched terms.

Moreover, comparative analysis of the top 30 categories for total gene expression regulation in the control and GnRHa-treated groups was performed. GO analysis was used to examine the enrichment of three categories in the total of 20,030 unigenes: biological processes, cellular components, and molecular functions.

**Fig. 3:**
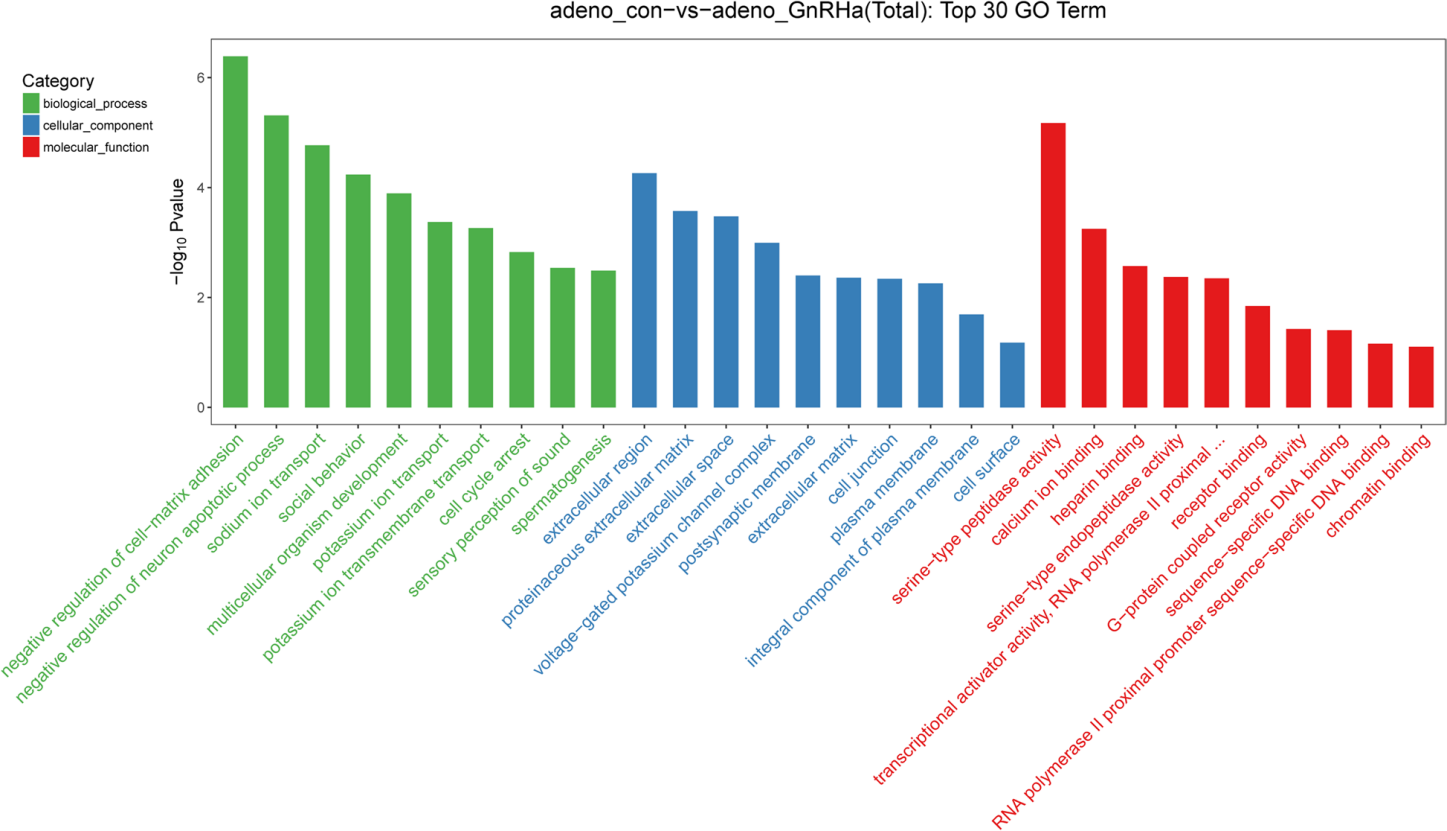
Comparative analysis of the top 30 categories enriched in DEGs between the control and GnRHa-treated groups. The DEGs are arranged into three functional categories: biological processes, cellular components, and molecular functions

For the biological process category, the most dominant subcategories were proteolysis, cell redox homeostasis, and the defense response to bacteria, followed by Kupffer’s vesicle development and others. For the cellular component category, the extracellular space and ciliary basal body were the most dominant subgroups. For the molecular function category, unigenes were assigned to serine-type endopeptidase activity, peptidase activity, serine-type peptidase activity, and metallocarboxypeptidase activity (Fig. 3).

### Verification of mRNA expression levels

**Fig. 4:**
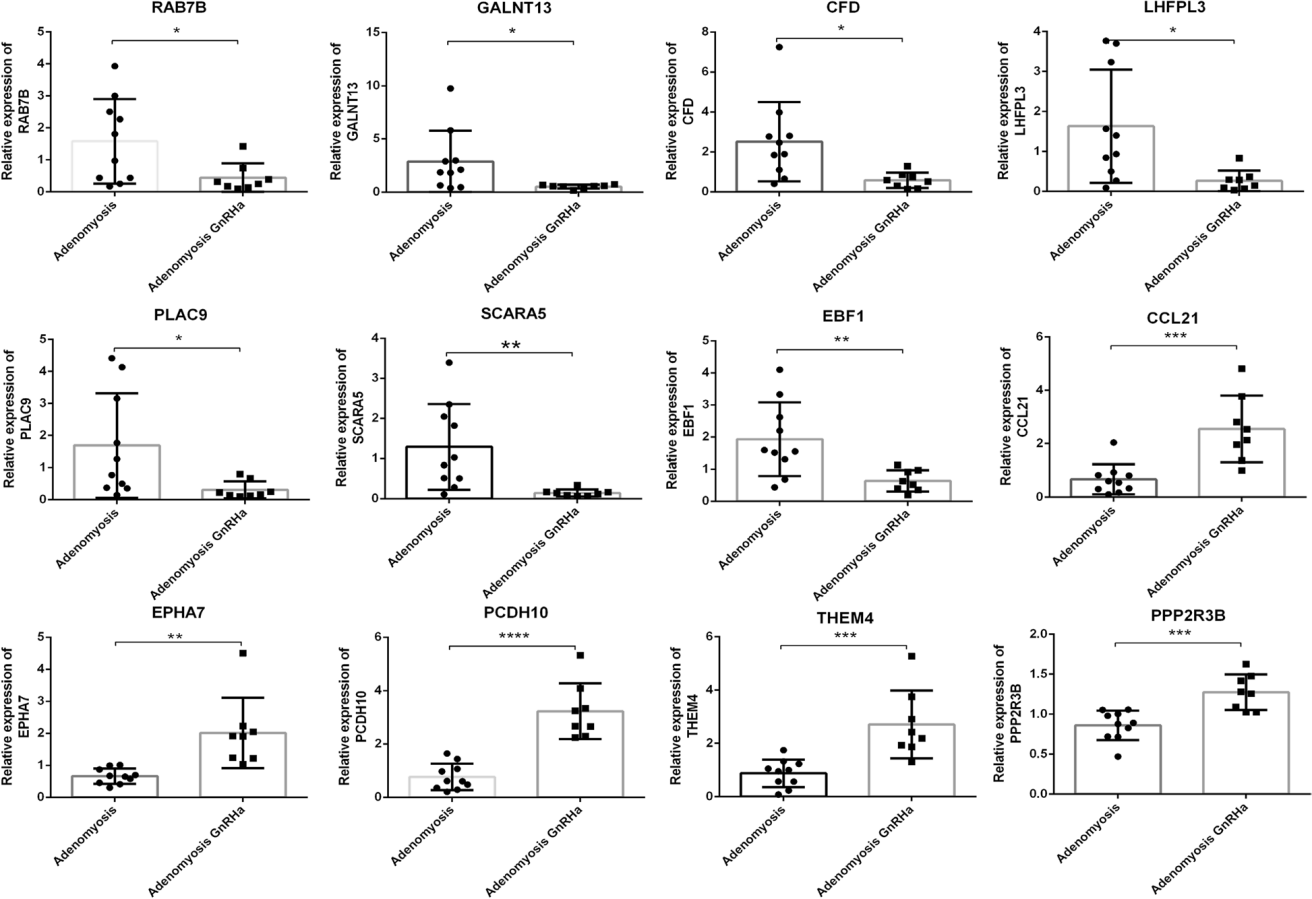
Verification of gene expression by real-time PCR in GnRHa-treated (*n* = 8) or untreated (*n* = 10) endometrium from patients with adenomyosis. (*P < 0.05, **P < 0.01, ***P < 0.001, statistically significant by Student’s t test)

To confirm the accuracy of the RNA-Seq results, 12 upregulated and downregulated genes were selected for further verification. Total RNA was extracted from adenomyotic endometrium with or without GnRHa treatment and used to verify the expression levels of the selected genes by qPCR (Fig. 4). The verification results were consistent with the RNA-Seq results, which suggests that the RNA-Seq results are accurate.

### Immunohistochemical analysis of CCL21

**Fig. 5:**
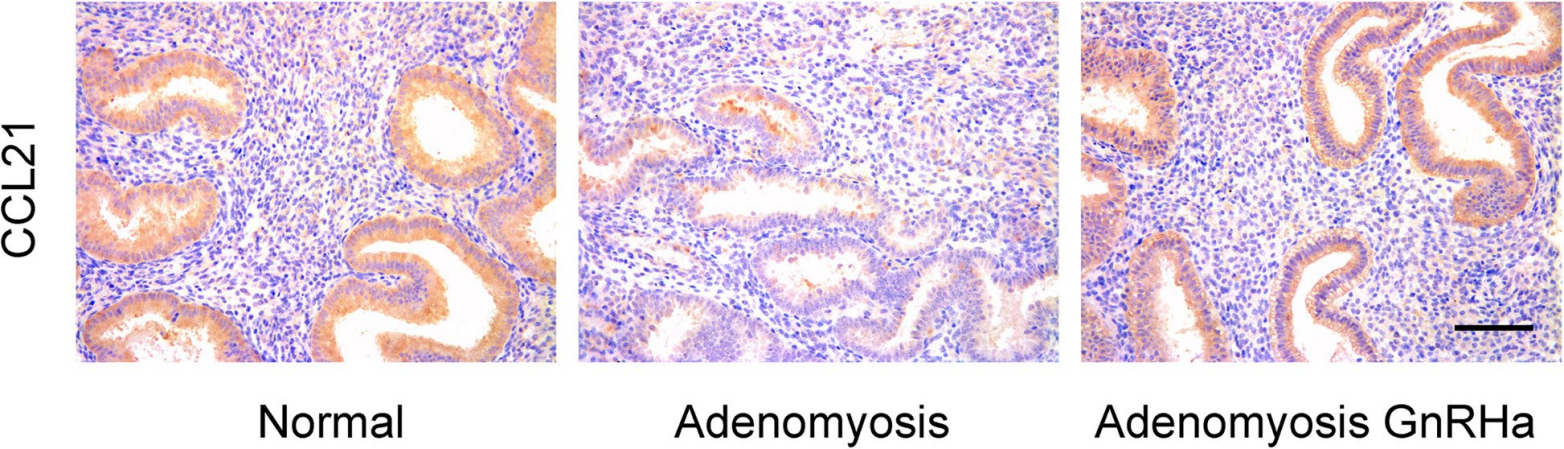
Representative immunohistochemical micrograph of CCL21 in different endometrial tissues (scale bar = 100 μM)

Through KEGG pathway classification, we found that the DEGs are mainly enriched in environmental information processing and organismal systems. Among the enriched KEGG pathways, environmental information processing was further divided into signaling molecules and interactions and signaling transduction, and organismal systems included the immune system, sensory system and nervous system (Table 2). CCL21 was noted among signaling molecules and interactions, signal transduction and the immune system, suggesting its potential role in the mechanism of GnRHa in the treatment of adenomyosis (Table 2). To identify chemokines that are differentially expressed in glands from the eutopic endometrium of patients with adenomyosis before and after GnRHa treatment, we collected endometrium from normal control patients and patients with adenomyosis before and after GnRHa treatment for immunohistochemical analysis. The expression of CCL21 was observed in the endometrium at the mid-secretory phase, especially in glandular epithelial cells, while it was significantly decreased in the endometrium of patients with adenomyosis (Fig. 5). After 3–6 months of GnRHa treatment, the expression of CCL21 in the endometrium of adenomyosis patients increased to normal levels, consistent with our RNA-Seq and qPCR results. Therefore, the expression of CCL21 was decreased in patients with adenomyosis, and GnRHa treatment upregulated the expression of CCL21 in patients with adenomyosis. These results suggest that CCL21 plays an important role in adenomyosis.

**Table 2:** Functional analysis for the differentially expressed genes in endometrium from women with adenomyosis before and after GnRHa treatment

Classification	Term	DEGs
Environmental information processing	Signaling molecules and interaction	AVPR1B; **CCL21**; CCR8; GABRB2; GRIA3; HTR1B; HTR2A; HTR2C; IL34; NGF; PTGER3
	Signaling transduction	ATP1A2; AVPR1B; **CCL21**; FST; GRIA3; HTR1B; HTR2A; HTR2C; MS4A2; NGF; NR4A1; PDGFD; PPP2R3B; PRKG2; PTGER3; PTPN5; SERPINE1; SFRP5; THEM4
Organismal systems	Immune system	**CCL21**; CCR8; CFD; F5; GRK1; MS4A2; PRKG2; SERPINE1
	Sensory system	GRK1; HTR1B; HTR2A; HTR2C; NGF; PRKG2; SCN3A; SCNN1G; TAS2R1; TAS2R31
	Nervous system	GABRB2; GRIA3; HTR1B; HTR2A; HTR2C; KCNQ3; NGF; PPP2R3B; PRKG2; SLC17A8; TH; UNC13C

### Examination of CCL21 function in adenomyosis in mice

**Fig. 6:**
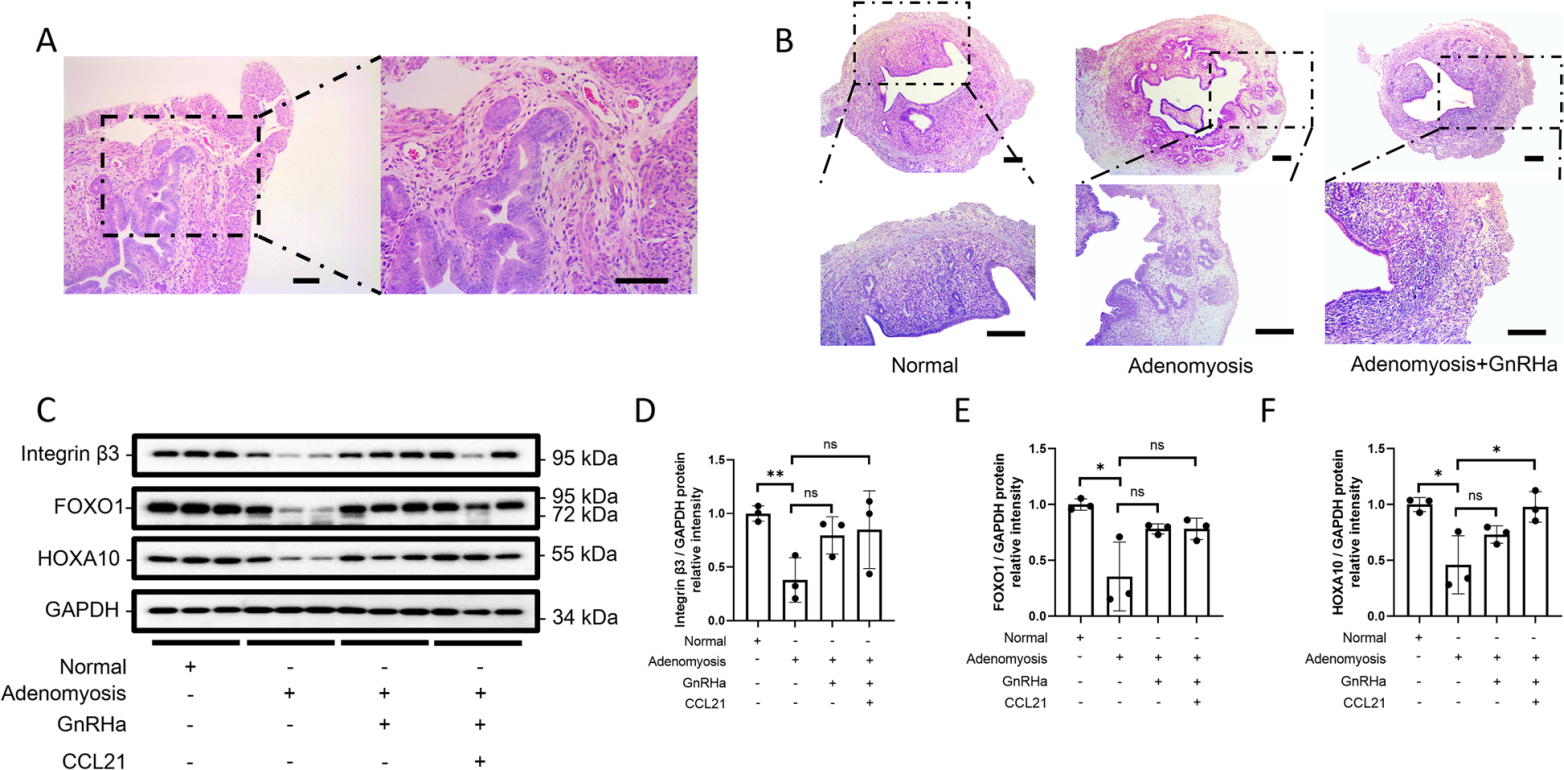
The function of CCL21 and GnRHa in mice with adenomyosis. **A**, HE staining of the uterus of model mice with adenomyosis (scale bar = 100 μM). **B**, Representative HE staining micrograph (scale bar = 200 μM) of the uterus from a normal mouse (Normal) and model mice with adenomyosis with or without GnRHa treatment (Adenomyosis; Adenomyosis with GnRHa). **C**, Integrin β3, FOXO1 and HOXA10 protein levels in the uterus in four groups: normal mice, adenomyosis mice, adenomyosis mice with GnRHa and adenomyosis mice with GnRHa+CCL21 treatment. **D-F** Total protein levels were normalized to total GAPDH levels in the western blot analysis (*P < 0.05, *n* = 3, statistically significant by Student’s t test)

Guo et al. found that GnRHa, as a clinical treatment for adenomyosis, can also improve endometrial receptivity in adenomyosis model mice [10]. To further study the role of CCL21 in adenomyosis, we generated model mice with adenomyosis and treated them with GnRHa. Gland invasion and muscle disorder in the uterus of mice with adenomyosis were observed though HE staining (Fig. 6A). In the uterus of mice with adenomyosis after GnRHa treatment, markers of adenomyosis were alleviated (Fig. 6B) and the expression of receptivity markers increased although there was no statistical difference (Fig. 6C-F). CCL21 can regulate the recruitment of Treg cells, which affect the receptivity of the endometrium in adenomyosis [11]. After combined administration of GnRHa and CCL21 in the uterus of mice with adenomyosis, expression of the endometrial receptivity marker HOXA10 was significantly increased (Fig. 6C, F). The addition of CCL21 may further promote the therapeutic effect of GnRHa on adenomyosis, thereby improving endometrial receptivity.

## Discussion

Adenomyosis is detrimental to the clinical outcome of ART [12]. Long-term GnRHa pretreatment promotes embryo implantation and reduces the miscarriage rate [13]. Khan et al. found that GnRHa could reduce inflammation and angiogenesis and inhibit the proliferation and induce the apoptosis of cells derived from ectopic tissue [7, 14]. The clinical outcome of patients with RIF improved after they received GnRHa and hormone replacement therapy (G-HRT), which may be associated with an increase in endometrial receptivity [15].

Through RNA-Seq analysis, this study demonstrates that GnRHa treatment altered the gene expression profile of the eutopic endometrium in adenomyosis. Functional assessment through KEGG pathway analysis illustrated that signal transduction and organismal systems (nervous system, sensory system and immune system) changed significantly upon GnRHa treatment. Signal transduction involves the transmission of biological signals between cells and plays important roles in cell division, differentiation, metabolism and death. Pain is a common symptom of adenomyosis caused by inflammation and associated with abnormal innervation [16]. The autonomic nervous system is distributed in the female reproductive system, where it regulates blood vessels, smooth muscle contraction and neuroendocrine function [17]. Many studies have shown that the imbalance between sympathetic and sensory innervation and subsequent abnormal secretion of cytokines are involved in the neurogenesis of adenomyosis and lead to peripheral nerve inflammation [18]. Lymphocytes and macrophages are increased in the endometrium of patients with adenomyosis, accompanied by abnormal expression of anti-inflammatory and proinflammatory cytokines [19]. Immune abnormalities are associated with epithelial-mesenchymal transition, which facilitates the migration of endometrial cells [20].

Hormonal treatment options could also have a direct effect on local immunity in the endometrium in addition to their systemic effect. Khan et al. found that the use of a preoperative GnRH agonist (gonadotropin-releasing hormone agonist, GnRHa) in patients with adenomyosis induced a decrease in MCP-1 levels and a reduction in the number of macrophages in endometrial and myometrial layers compared to those in untreated patients [7].

Chemokines have been suggested to participate in a series of pathophysiological processes, including cancer metastasis, angiogenesis and epithelial barrier formation [21–23]. Numerous studies have demonstrated that chemokines are abnormally expressed in the endometrium of patients with endometriosis and adenomyosis. The levels of CXCL12 and its receptor, chemokine (C-X-C motif) receptor 4 (CXCR4), were found to be significantly increased in both eutopic and ectopic endometrium from patients with adenomyosis [24]. The peritoneal fluid and serum of patients with endometriosis and adenomyosis were found to have elevated levels of inflammatory cytokines, including IL-6, IL-8, IL-1b, IFN-γ, and TNF-α, which was associated with natural killer (NK) cell dysfunction [25].

CCL21 is a decoy receptor ligand of chemokines or cytokines, and its dysregulation was reported to be associated with fetal loss through the mediation of increased inflammation in pigs [26]. CCL21 was also reported to take part in the inflammatory reaction in adenomyosis [27]. In addition, microarray analysis showed that CCL21 was upregulated in RIF patients, demonstrating that CCL21 may contribute to impaired endometrial receptivity [28], which suggests that abnormally elevated CCL21 might lead to excessive inflammation and ultimately implantation failure. At the same time, our study has revealed that CCL21 is downregulated in the endometrium of adenomyosis patients. KEGG pathway analysis illustrated the involvement of CCL21 in signaling molecules and interactions, signal transduction and the immune system (Table 2), which suggests the potential influence of CCL21 dysregulation in adenomyosis.

To ensure a conducive immune environment for embryo implantation, maternal immune tolerance mediated by CD4+ Tregs is essential [29]. Tregs support the adaptation of maternal blood vessels through immune regulation and inhibit inflammation, thereby promoting the invasion of trophoblasts [30, 31]. Tregs have been shown to express CCR7 and migrate under the chemotactic effect of CCL21 [32]. Abnormal expression of chemokines in the endometrium may affect embryo implantation and cause infertility in patients with adenomyosis [24]. The expression levels of Tregs are decreased in the peripheral circulation and uterus of patients with adenomyosis [11]. The number of Tregs is decreased in patients with repeated implantation failure and increased after GnRHa treatment, suggesting that GnRHa may have an immunomodulatory effect and improve endometrial receptivity [33]. CCL21 levels were found to increase in the endometrium of patients with adenomyosis after GnRHa treatment, which may affect the outcome of pregnancy by regulating the recruitment of Tregs. The RNA-Seq data provide new evidence for the regulation of chemokines in the endometrium of patients with adenomyosis after GnRHa treatment. A series of studies have shown that several chemokines are expressed in the eutopic and ectopic endometrium of patients with adenomyosis. Some of them exert a proinflammatory function, while others have anti-inflammatory functions. Monocyte chemoattractant protein-1 (MCP-1)/chemokine (C-C motif) ligand 2 (CCL2) plays important roles in regulating monocyte/macrophage migration and recruiting monocytes to the site of inflammation after tissue damage or infection [34]. The cytokine IL10, which has potential anti-inflammatory effects, has been found to be reduced in expression in the endometrium of patients with adenomyosis [35, 36]. Therefore, to a certain extent, the expression levels of chemokines and their receptors reflect the recruitment of immune cells and the regulation of inflammation in the endometrium after adenomyosis and GnRHa treatment.

The expression level of CCL21 was confirmed through RNA-Seq analysis and further validation, and its potential function was predicted through KEGG analysis and assessment of common chemokine function in adenomyosis. CCL21 was suggested to regulate the endometrial immune environment by recruiting Tregs. Our results indicate that when CCL21 and GnRHa were combined to treat mice with adenomyosis, they enhanced the expression of HOXA10 to promote endometrial receptivity. Meanwhile, CCL21 may also affect the communication between cells by participating in cell signal transduction and interactions. One focus of our next research will be determining whether CCL21 can regulate the endometrial immune environment by recruiting Tregs in adenomyosis and further improve the receptivity of the endometrium. We also look forward to further clarifying the specific molecular mechanisms involved in signal transduction and interactions regulated by CCL21.

## Conclusions

Through RNA-Seq analysis of the eutopic endometrium from GnRHa-treated or untreated patients with adenomyosis, we identified 132 DEGs related to immune system-associated signal transduction; these DEGs are predicted to regulate immune regulation of the uterine local microenvironment and therefore affect endometrium receptivity. The immunoreactive chemokine CCL21 was found to be highly expressed after GnRHa treatment, and CCL21 combined with GnRHa is suggested to promote endometrial receptivity in adenomyosis.

## Data Availability

The datasets used and/or analysed during the current study are available from the corresponding author on reasonable request.

## Abbreviations

ART: Assisted reproductive technology
GnRHa: Gonadotropin releasing hormone agonists
RNA-Seq: RNA sequencing
DEGs: Differentially expressed genes
CCL21: Chemokine (C-C motif) ligand 2
IVF-ET: In vitro fertilization-embryo transfer
FET: Frozen-embryo transfer
RIF: Recurrent implantation failure
MRI: Magnetic resonance imaging
FPKM: Fragments per kilobase per million
DPC: Day post coitus
PCA: Principle component analysis
KEGG: Kyoto encyclopedia of genes and genomes
G-HRT: GnRHa and hormone replacement therapy
CXCL12: Chemokine (C-X-C motif) ligand 12
CXCR4: Chemokine (C-X-C motif) receptor 4
MCP-1: Monocyte chemoattractant protein-1
